# Draft genome sequence of *Sphingomonas* sp. GlSt437 isolated from the Styx Glacier, Antarctica

**DOI:** 10.1128/mra.00246-25

**Published:** 2025-08-11

**Authors:** Minkyung Kim, Subin Lee, Ok-Sun Kim

**Affiliations:** 1 Division of Life Sciences, Korea Polar Research Institute 123591, Incheon, Republic of Korea; Indiana University Bloomington 1771, Bloomington, Indiana, USA

**Keywords:** *Sphingomonas*, genomic analysis, Antarctica

## Abstract

We present the draft genome of *Sphingomonas* sp. GlSt437 isolated from the Styx ice core, Antarctica. The genome of strain GlSt437 comprises 3,409,635 bp with a GC content of 64.9%, encoding 3,274 genes.

## ANNOUNCEMENT

The genus *Sphingomonas* is a gram-negative, aerobic, yellow-pigmented bacterium isolated from diverse environments, including tap water (1), soil (2), marine bivalves (3), freshwater ponds (4), and plant roots (5). Genomic analyses have revealed the metabolic versatility of *Sphingomonas*, including its capacity to produce metabolites such as phytohormones, indole-3-acetic acid, and nostaxanthin, as well as its ability to degrade environmental pollutants (6, 7). The ubiquitous distribution of *Sphingomonas* in nature highlights the significance of genome elucidation for understanding its ecological adaptations to specific environmental niches. Here, we report the genome sequences of *Sphingomonas* sp. GlSt437 isolated from 10 m depth of the Styx ice core, Antarctica (73° 51.10′S, 163° 41.22′E).

Strain GlSt437, isolated from a 10 m depth ice core collected from Styx Glacier (8), was incubated at 10°C for 2 months on 0.01× nutrient agar (BD Difco, USA). Colonies were isolated and streak-purified on fresh plates three times. The genomic DNA of strain GlSt437 was extracted with the Qiagen MagAttract HMW DNA Kit (Qiagen, Hilden, Germany) after harvesting colonies from three individual plates, according to the manufacturer’s protocol. The genome of strain GlSt437 was sequenced using Illumina MiSeq and PacBio Sequel. The DNA concentration was measured using a Qubit 2.0 fluorometer (Invitrogen, Carlsbad, USA). For Illumina sequencing, genomic DNA was sheared to approximately 550 bp using an ultrasonicator^TM^ (Covaris Ltd, Brighton, UK). The library was prepared with the TruSeq DNA Library LT kit (Illumina, San Diego, USA). Raw Illumina reads were quality-trimmed using Trimmomatic v0.36, and PhiX control sequences were filtered out using BBMap v38.32. For PacBio sequencing, genomic DNA (5 µg) was sheared using the Megaruptor 3. An SMRTbell library was constructed using the SMRTbell Express Template Preparation Kit (PacBio, Cat No. 100-938-900). Genome size, library fragment size, and Qubit concentration were entered into the PacBio library pooling calculator, and samples were pooled accordingly. DNA below 3 kb was excluded, and sequencing primers and polymerase were removed by AMPure XP bead purification (Beckman Coulter, USA), following the manufacturer’s instructions. Sequencing was performed on the PacBio Sequel system with the Sequel Sequencing Kit v.3.0 and SMRT Cell 1M v.2, using one 10 h movie per cell. A hybrid assembly was conducted with Unicycler version 0.5.0, utilizing quality-filtered MiSeq reads and PacBio long-read data (9). Default parameters were used except where otherwise noted.

The draft genome of strain GlSt437 was composed of six contigs with a maximum length of 1,917,613 bp, and the total genome size was 3.4 Mb with a G + C content of 64.9% (Table 1). Among the six contigs, contig 4 (73,869 bp) was assembled in a circular form and was further predicted to be a plasmid based on analyses using both PlasClass and Platon (10, 11). Protein-coding sequences were predicted by Prodigal 2.6.2. Among the encoding sequences of the entire genome, 3,031 (97.3%) genes were categorized using the GenoVi tool (12). The genes of the genome in strain GlSt437 were classified into 21 categories, mainly including cell wall/membrane/envelope biogenesis (7.2%), transcription (6.8%), post-translational modification, protein turnover, chaperones (6.1%), and amino acid transport and metabolism (6.0%).

**TABLE 1:** Genomic features of *Sphingomonas* sp. GlSt437

	*Sphingomonas* sp. GlSt437
Illumina MiSeq	
Length of raw reads	1,600,502,227 bp
Number of raw reads	5,426,018
N50	301 bp
PacBio Sequel	
Length of raw reads	3,193,141,699 bp
Number of raw reads	485,550
N50	8,219 bp
Hybrid assembly	
Size	3,409,635 bp
N50	1,917,613 bp
G + C content	64.89%
Number of predicted CDSs^*a*^	3,274
Number of rRNA genes	1, 1, and 1 (5S,16S, and 23S)
Number of tRNAs	43

^*a*^ Coding sequences.

## Data Availability

The genome sequence of Sphingomonas sp. GlSt437 is deposited in the GenBank database under the accession numbers JBPGYA000000000 with BioProject PRJNA1203301. The raw reads are deposited in the Sequence Read Archive (SRA) under the accession numbers SRR32573194 for Illumina sequencing and SRR32573789 for PacBio sequncing.
